# Comparison of Sampling Designs for Estimating Deforestation from Landsat TM and MODIS Imagery: A Case Study in Mato Grosso, Brazil

**DOI:** 10.1155/2014/919456

**Published:** 2014-09-01

**Authors:** Shanyou Zhu, Hailong Zhang, Ronggao Liu, Yun Cao, Guixin Zhang

**Affiliations:** ^1^School of Remote Sensing, Nanjing University of Information Science & Technology, Nanjing 210044, China; ^2^Institute of Geographic Sciences and Natural Resources Research, CAS, Beijing 100101, China

## Abstract

Sampling designs are commonly used to estimate deforestation over large areas, but comparisons between different sampling strategies are required. Using PRODES deforestation data as a reference, deforestation in the state of Mato Grosso in Brazil from 2005 to 2006 is evaluated using Landsat imagery and a nearly synchronous MODIS dataset. The MODIS-derived deforestation is used to assist in sampling and extrapolation. Three sampling designs are compared according to the estimated deforestation of the entire study area based on simple extrapolation and linear regression models. The results show that stratified sampling for strata construction and sample allocation using the MODIS-derived deforestation hotspots provided more precise estimations than simple random and systematic sampling. Moreover, the relationship between the MODIS-derived and TM-derived deforestation provides a precise estimate of the total deforestation area as well as the distribution of deforestation in each block.

## 1. Introduction

Human-induced and natural forest disturbances change forest systems by influencing their composition, structure, and functional processes [1]. Deforestation is the conversion of forested areas to nonforest land uses, such as arable land, urban areas, logged areas, or wasteland [2], and is important for forest resource management, biodiversity conservation, climate change, the global carbon cycle, and sustainability management [3–6]. Research on the accurate monitoring of deforestation and its influence is a topic of considerable interest in the context of global warming.

Remotely sensed data with coarse spatial resolution, such as the Advanced Very High Resolution Radiometer (AVHRR) and Moderate-resolution Imaging Spectroradiometer (MODIS), are commonly used over large areas, such as national, continental, climate zone, or global scales [6–8]. Because most deforestation occurs at subpixel scales [9, 10], these data are inadequate for directly and precisely estimating deforestation [11]. High spatial resolution data, such as Landsat data, allow for more accurate quantification of deforestation areas [12–14]. However, the infrequent repeat coverage, frequent cloud cover, and data costs often preclude the use of wall-to-wall mapping approaches with Landsat data for monitoring long-time deforestation over large regions [6, 15, 16]. Most research adopts sample-based methodologies to estimate deforestation with higher spatial resolution imagery [6, 10, 13, 17]. Based on the estimated results from the sampling regions, the deforestation area or even the distribution of deforestation of the entire study area can be extrapolated.

Sample-based methods that use a probability sampling design provide a quantitative measure of the precision of the uncertainty that is attributable to sampling and construct confidence bounds for the area of deforestation. The precision depends on the number of samples and their locations. Sampling is a cost- and time-efficient alternative if the objective is to estimate the area of deforestation rather than to map deforestation. Sample-based methodologies can be classified as random sampling, stratified sampling, and systematic sampling. The random sampling method randomly selects complete images or several small blocks within the area of interest and then analyzes the deforestation [6]. This technique has been applied by lots of applications [15–19]. Based on parameters such as biome, precipitation, elevation, dominant forest types, land cover types, disturbance degree, topography, and soil types, the stratified sampling method divides the study area into several strata and then selects the same number of samples or allocates more samples into the strata with greater expected levels of deforestation [20, 21]. The stratified sampling strategy is the common used method in areas such as humid tropical forests [10, 22–24]. Systematic sampling selects samples at a defined spatial interval and is easily performed compared to the two sampling methods described above. Systematic sampling designs have been adopted to monitoring deforestation over large areas by numerous researchers [13, 14].

Deforestation is a complex phenomenon of forest cover change that is usually unevenly distributed and often converges within a smaller region [18, 25, 26]. Although some sampling method has been recommended for operational assessments of global and regional deforestation rates in the tropics [27] or has been implemented in several studies [10, 20, 21, 24], the sampling strategies have both advantages and disadvantages; sample-based methods for this specific application has only been the subject of limited evaluation [6]. Comparisons between different sampling designs are required to further illustrate the adaptability of the sampling method to various spatial and temporal scales. This paper compares and discusses the precisions of deforestation estimated by different sampling strategies using the state of Mato Grosso in Brazil as an example. Landsat imagery and a MODIS dataset from nearly the same period are used to identify areas of deforestation and select sample blocks within the forest cover regions.

## 2. Study Area and Data

### 2.1. Study Area

**Figure 1:**
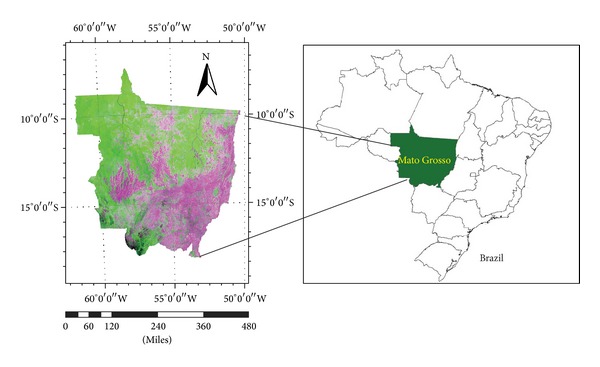
The study area of Mato Grosso (MT) in Brazil.

The study area of Mato Grosso (MT) is situated in the midwestern part of Brazil and is one of the states that have been designated as part of the Legal Amazon (Figure 1). MT's total land area is 903,386 km^2^, of which up to 67.8% is part of the southern basin of the Amazon River [28]. The primary vegetation is characterized by latitudinal variations from forests in the north to mixed zones in the middle to Cerrado in the south [29]. Deforestation in the Brazilian Amazon has been monitored for more than two decades using a variety of satellite sensors. Deforestation data products have identified periods of increasing (2001–2004) and decreasing (2005–2007) deforestation rates in Mato Grosso [30].

### 2.2. Data and Preprocessing

#### 2.2.1. Landsat TM

Based on the characteristics of the study area, the high spatial resolution (30 m × 30 m) multispectral satellite sensor Landsat Thematic Mapper (TM) is selected to assess the distribution of deforestation. This deforestation dataset is used as a reference for evaluating different sampling strategies. Forty-eight Landsat TM scenes from paths 223 to 231 and rows 65 to 72 were obtained during dry periods (times of vegetation growth) from June, 2005, to August, 2006. All of the TM image data were preprocessed with radiometric calibration, atmospheric correction based on the 6S radiant transfer model, geometric correction and registration, and image mosaicking.

#### 2.2.2. MODIS

The MODIS imagery dataset is composed of the 8-day composite 1 km daytime land surface temperature (LST) dataset MYD11A2, the 16-day composite 500 m enhanced vegetation index (EVI) dataset MYD13A1, and the yearly 500 m land cover type dataset MCD12Q1 from 2005. The LST data and EVI data were imaged from June 10, 2002, to August 15, 2006, in which the data obtained from June, 2005, to August, 2006, (the period of being evaluated for deforestation) corresponded to the same period as the TM data and the other data were needed as the reference in the deforestation detection algorithm. The EVI data were used because saturation levels are avoided, whereas the normalized difference vegetation index (NDVI) tends to approach saturation levels in high biomass regions, which has important consequences for change detection [31]. The MODIS land cover is used to mask all nonforest pixels, leaving all forest type pixels classified as evergreen needleleaf forests, evergreen broadleaf forests, deciduous needleleaf forests, deciduous broadleaf forests, and mixed forests. All of the deforestation detection processes are focused on the forest cover pixels. The preprocessing steps for the MODIS data include resampling the LST data to a spatial resolution of 500 m, mosaicking, projection conversion, quality control to remove pixels with cloud contamination or low quality, and creating subsets using the vector data of MT.

#### 2.2.3. PRODES

The Brazilian National Institute for Space Research (INPE) PRODES has provided annual wall-to-wall deforestation maps of the Brazilian Legal Amazon [32] since 2000. These maps are used to evaluate the precision of the deforestation detected from the Landsat and MODIS imagery. PRODES employed a linear spectral mixing algorithm to generate vegetation, soil, and shade fraction images. The soil and shade fraction images were classified using image segmentation, followed by unsupervised classification, image editing, and mosaicking. PRODES pixels that had been interpreted as deforestation by the PRODES analysts between the study periods were marked as deforestation, and pixels interpreted as other PRODES classes were labeled as no-change.

## 3. Methodology

**Figure 2:**
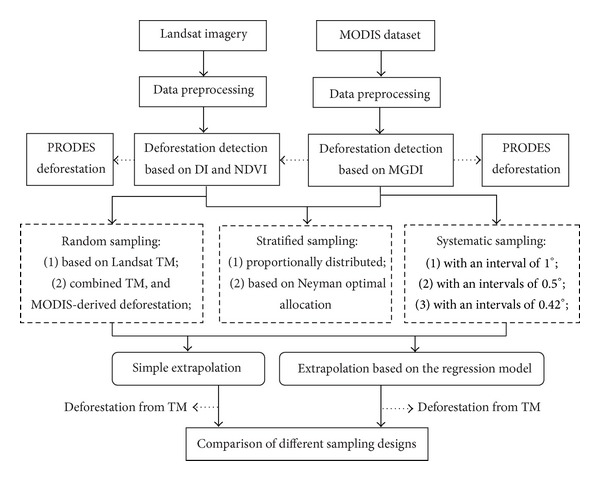
Research methodology flowchart.

A flowchart of the research methodology is shown in Figure 2.

### 3.1. Deforestation Detection from Landsat TM Data

The disturbance index (DI) is a simple and effective means of tracking vegetation disturbance across a variety of forest ecosystems and is especially useful for identifying complete forest canopy removal [33, 34]. The DI is based on the Tasseled-Cap data space. The DI algorithm assumes that a combination of the greenness and the wetness can highlight the spectral response characteristics of the vegetation, while the brightness can express the characteristics of nonvegetation areas. The brightness values of disturbed areas are higher than those of nondisturbed forest areas, while the greenness and wetness values are lower [33].

At a basic level, the DI records the normalized spectral distance of any given pixel from a nominal “mature forest” class to a “bare soil” class. The DI is calculated using the Tasseled-Cap indices for Landsat TM/ETM+ [34]:

(1) $$\begin{array}{c} \text{DI}=B^{\prime }-\left(G^{\prime }+W^{\prime }\right), \end{array}$$

where *B*′, *G*′, and *W*′ represent the Tasseled-Cap brightness, greenness, and wetness indices, respectively, normalized by a dense forest class for each Landsat scene, such as

(2) B′=B−uBσ  B  ,

where *μ*_*B*_ is the mean of the Tasseled-Cap brightness index and *σ*_*B*_ is the standard deviation of brightness within the dense forest class for a particular scene.

Because the DI values are based on the statistics of the forest reflectance from individual scenes, the DI metric is relatively insensitive to the variability in solar geometry or the bidirectional reflectance distribution function (BRDF) between scenes and lessens the effect of vegetation phenological variability between image dates [34].

The original application by Healey et al. [33] relied on the absolute value of the DI from individual scenes to assess the extent of disturbance. Masek et al. [34] used the decadal change in DI value (ΔDI) as a more robust metric to identify disturbance and recovery. In this study, we combined the absolute value of DI_2005_ of the initial stage, the change in DI value, and the change in NDVI to detect deforestation from the TM imagery. The ΔDI and ΔNDVI were calculated as the temporal change DI_2006_-DI_2005_ and NDVI_2006_-NDVI_2005_, respectively. DI values greater than 1.0 have a high probability of being disturbed or nonforest. Large positive values of ΔDI or ΔNDVI correspond to likely disturbance events, and ΔDI × ΔNDVI can increase the pixel difference between deforestation and nondeforestation. Thresholds were applied to the DI_2005_ and ΔDI × ΔNDVI values to identify potential deforestation (DI_2005_ < thresh1 and ΔDI × ΔNDVI > thresh2).

### 3.2. Deforestation Detection from MODIS Data

The MODIS global disturbance index (MGDI) algorithm [35, 36] was adopted for deforestation mapping from the LST and EVI data. The MGDI algorithm has been successfully tested over the Western US and North America.

Deforestation caused by instantaneous disturbances, such as wildfire, results in an immediate departure of the LST/EVI ratio from the range of natural variability [36], which can be detected by MGDI_Inst_ as defined by

(3) $$\begin{array}{c} \text{MGD}{\text{I}}_{\text{Inst}}=\frac{{\left(\text{LS}{\text{T}}_{\max}/\text{EV}{\text{I}}_{\max\_\text{post}}\right)}_{y}}{{\left(\text{LS}{\text{T}}_{\max}/\text{EV}{\text{I}}_{\max\_\text{post}}\right)}_{y-1}}, \end{array}$$

where MGDI_Inst_ is the instantaneous MGDI value, *y* is the period (from June, 2005, to August, 2006) being evaluated for deforestation, (*y* − 1) is the period from June, 2002, to May, 2005. LST_max⁡_ is the maximum 8-day composite LST for the computation period and EVI_max⁡_post_ is the maximum 16-day EVI that occurred after the maximum LST during the same period.

Noninstantaneous disturbance events, such as hurricanes and insect epidemics, cause departures from the range of natural variability in the year following the disturbance [36]. The noninstantaneous variant of the MGDI algorithm is given as follows:

(4) $$\begin{array}{c} \text{MGD}{\text{I}}_{\text{Non-Inst}}=\frac{{\left(\text{LS}{\text{T}}_{\max}/\text{EV}{\text{I}}_{\max}\right)}_{y}}{\bar{{\left(\text{LS}{\text{T}}_{\max}/\text{EV}{\text{I}}_{\max}\right)}_{y-1}}}, \end{array}$$

where MGDI_Non-Inst_ is the noninstantaneous MGDI value and EVI_max⁡_ is the maximum 16-day composite EVI during the computation period.

When calculating MGDI_Inst_ and MGDI_Non-Inst_, for the numerator in (3) and (4), LST_max⁡_, EVI_max⁡_post_, and EVI_max⁡_ during the deforestation evaluated period *y* were determined for each pixel and then the LST/EVI ratio could be calculated. As for the denominator, the period from June, 2002, to May, 2005, was averagely divided into three parts and each subperiod included 12 months (from June to May of the next year); the LST_max⁡_, EVI_max⁡_post_, and EVI_max⁡_ for each subperiod were extracted to compute the LST/EVI ratio, and then the mean LST/EVI ratio for all times (*y* − 1) was determined for each pixel.

The difference between the instantaneous disturbances and the noninstantaneous disturbances is controlled by the rate of disturbance event. The MGDI is a dimensionless value, and a given pixel's MGDI value will tend toward unity in the absence of disturbance. When a major disturbance event, such as wildfire, occurs, LST will increase and EVI will decrease instantaneously, resulting in a MGDI value that is much larger than the multiyear mean. MGDI_Inst_ and MGDI_Non-Inst_ are combined to be used for continuous wall-to-wall deforestation detection in the research. Given a suitable threshold, the deforestation pixels can be determined based on the MGDI map.

### 3.3. Sampling Strategies Design

Tucker and Townshend [17] randomly sampled complete Landsat images and determined that using whole scenes will result in smaller standard errors and save little or nothing in acquisition costs. However, Duveiller et al. [13] suggested that the sampling efficiency can be increased significantly by using small image extracts as sampling units and having them systematically (rather than randomly) distributed over the forest domain. Even if the standard deviation of a small region is greater than that of a large region, more samples could compensate for the larger standard deviation, which means that more sampling units with small areas might achieve a higher precision [37–39].

Based on these analyses, we sampled small 15 km × 15 km blocks, and the entire study area was divided into 4081 blocks. The area and proportion of deforestation in each block corresponding to the Landsat TM and MODIS results could then be calculated. Three sampling strategies were designed to select sample sites to estimate the deforestation areas in MT during the period from 2005 to 2006. The difference between our sampling designs and those in previously published studies is that the selected sample blocks must include some forest pixels based on the MODIS MCD12Q1 data.

#### 3.3.1. Random Sampling

Two random sampling methods were used. First, we simply randomly selected samples within the forest cover region. However, because the deforestation regions usually distribute densely, sampling sites and the variation in densities could influence the precision of the results when using simple random sampling. Thus, we then selected samples within the regions where the proportion of deforestation from the MODIS dataset was greater than a threshold. Various deforestation proportion thresholds were used to further analyze the differences in the estimation results.

#### 3.3.2. Stratified Sampling

The stratification was based on the MODIS-derived deforestation. The resulting low, medium, high, and very high deforestation strata were defined as MODIS-derived deforestation proportions of 0-1%, >1–5%, >5–8%, and >10% per block, respectively. The sample sites were allocated to the four strata using two different methods. First, the samples are proportionally distributed. And second, we selected samples based on Neyman optimal allocation [6]. The optimal allocation was determined using per stratum variances of the MODIS-derived percentage of deforestation for all blocks within each stratum.

#### 3.3.3. Systematic Sampling

The systematic sampling design is based on the number of samples, and fixed intervals were used to obtain sample blocks. Furthermore, if there were no forest pixels for a certain selected block, we sampled the nearest block to the right or down as a substitute.

### 3.4. Deforestation Extrapolation and Precision Evaluation

Two methods were used to compare the extrapolation precision of the deforestation result over the entire study area. The first simple extrapolation method was based on

(5) $$\begin{array}{c} y=\frac{\sum_{i=1}^{n}x_{i}}{\sum_{i=1}^{n}S_{i}}\times A, \end{array}$$

where *y* is the estimated area of deforestation in the study area, *x*_*i*_ and *S*_*i*_ are the deforestation area and the forest area, respectively, for the *i*th sample block, and *A* is the total forest area of the study area.

Equation (5) can only be used to obtain the total deforestation area, but the distribution of deforestation cannot be spatially determined. To estimate the distribution of deforestation within each block, a second extrapolation method was adopted according to the relationship between the TM and MODIS deforestations derived from the sample blocks as in Hansen et al. [10]. A simple linear regression model (6) was used to estimate the Landsat-scale deforestation area in each block. The total deforestation in the study area is the sum of the deforestation in all of the blocks:

(6) $$\begin{array}{c} D_{\text{TM}}=a\times D_{\text{MODIS}}+b, \end{array}$$

where *D*_TM_ and *D*_MODIS_ are the corresponding deforestation areas in each block and *a* and *b* are the coefficients determined from the sample blocks.

Compared to the deforestation derived from the Landsat imagery, the relative error (*ε*) of the extrapolated deforestation results for the entire study area was evaluated with

(7) ε=|y−y  0  |y0×100%,

where *y* is the estimated deforestation area of the study area and *y*_0_ is the TM-derived deforestation area.

## 4. Results and Discussions

### 4.1. Deforestation Derived from TM Imagery and MODIS Data

Based on the PRODES deforestation data as the reference, by visually interpreting and comparing the TM color composite images pre- and postdeforestation, the thresholds used for detecting the deforestation from TM imagery were determined after several experiment tests. The pixels were preliminarily classified as deforested if their ΔDI × ΔNDVI values were greater than 5.0 and the DI_2005_ values were less than 20.0. Furthermore, each pixel was flagged as deforested using a median filtering method with a 3 × 3 pixel window to eliminate isolated pixels.

**Figure 3:**
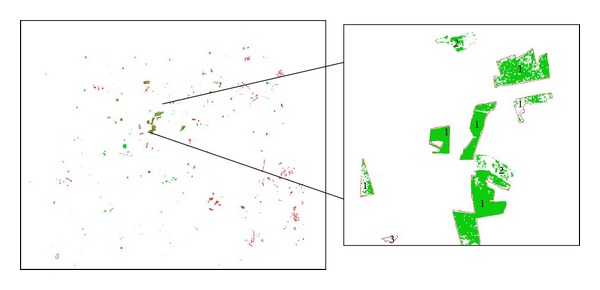
A comparison of the deforestation detected from the TM imagery (green areas) and the PRODES deforestation data (red polygons).

In the TM deforestation results, all of the nondeforestation and no-data pixels were masked out in white, and the deforestation pixels were displayed in green. The PRODES deforestation data were overlaid on the TM deforestation results to determine the detection precision. In general, the deforestation detected based on the TM imagery is spatially consistent with that determined from the PRODES data.  Figure 3 compares the deforestation detected from the TM imagery (path 226, row 69) and the PRODES deforestation vector data. The deforestation detected from the Landsat TM data was consistent with that from the PRODES data, such as at site 1. Some locations, such as site 2, were only detected from the TM data, while site 3 was only identified from the PRODES data.

**Figure 4:**
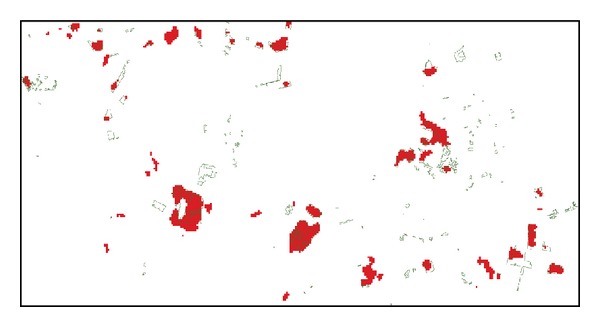
A comparison of the deforestation detected from the MODIS data (red areas) and the PRODES deforestation data (green polygons).

The MGDI algorithm described above was applied to the MODIS data for the years 2002–2006 to detect deforestation during the period from 2005 to 2006. The thresholds for detecting deforestation from the MGDI map were determined using the deforestation map derived from the TM data as the reference. We selected six deforestation areas where the disturbance information could be identified in MODIS data and tested different thresholds from 40% to 80% with the interval 5% increases from the multi-sub-periods mean value. Based on the calculation of total deforestation area across the selected area at each threshold, any pixels with MGDI values greater than 70% of the multiyear mean for instantaneous disturbance or greater than 50% of the multiyear mean for noninstantaneous disturbance were flagged as deforestation. The PRODES deforestation data were also overlaid on the MODIS deforestation results.  Figure 4 illustrates the precision of the MODIS deforestation results compared to the PRODES results. The large deforestation sites were successfully detected based on the MGDI algorithm.

The differences in the deforestation distributions generated from the TM imagery, MODIS data, and PRODES data were primarily caused by two factors. First, the TM and MODIS imagery were not collected at exactly the same time as the PRODES data. Some of the orbits were more than one month apart, and new deforestation might have occurred during the intervening period that could not be detected in both this study and the PRODES results. Second, the PRODES deforestation data represent only the loss of intact forest area (old growth forest), so the target parameter does not include areas of forest cover change due to degradation, regeneration, afforestation, regrowth, or clearing of regrowth. However, several sites in which deforestation occurred before 2005 as well as between 2005 and 2006, such as site 2 in Figure 3, were detected in this study.

**Figure 5:**
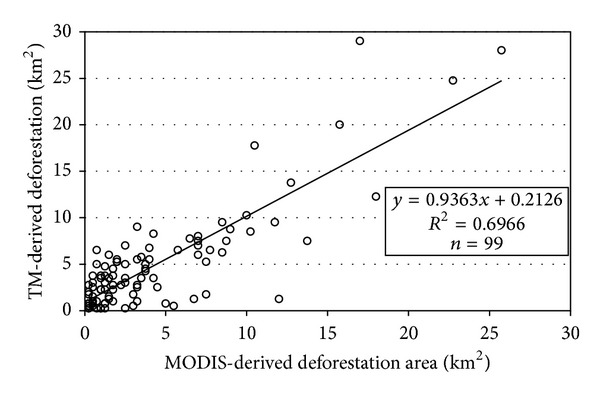
Plot of MODIS-derived versus TM-derived deforestation per sample block.

Because of the nearly identical image times of the MODIS and TM data, the deforestation results derived from the MODIS dataset and the TM imagery were compared by calculating the deforestation area within 99 15 k × 15 km blocks (Figure 5). There is a highly linear correlation between the MODIS-derived and the TM-derived deforestations, though the area derived from the TM data is slightly larger than that of MODIS.

### 4.2. Comparison of Sampling Designs Based on Simple Extrapolation

**Figure 6:**
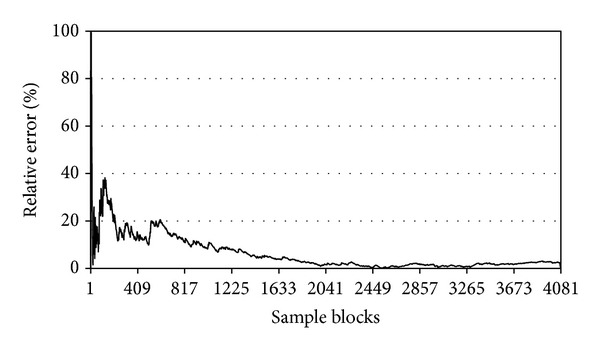
The variation of deforestation estimation error with the number of sample blocks.

We analyzed the influence of the number of sampling blocks on the estimation of deforestation using (5). The analysis began with three randomly selected blocks and increased to the total number of blocks in the study (4081), and each block could only be used once. The variation of the estimation error with the number of sampled blocks, as calculated with (7), is shown in Figure 6.

Figure 6 shows that the sample locations and the sampling density have a large effect on the estimation results. If the first sampling blocks are located at a site where a very small or a very large proportion of deforestation has occurred, the relative error will fluctuate until it reaches a stable value. Moreover, the estimation error varies little above a certain number of sampling blocks. To further illustrate the influence of the sampling density, we compared various sampling designs using 100, 350, and 500 sampling blocks.

#### 4.2.1. Error Analysis of Random Sampling Estimation

The random sampling technique was designed to randomly select sample blocks across the study area and in the blocks where MODIS-derived deforestation proportion is greater than 0.1%, 0.5%, and 1%.  Table 1 shows the relative estimation errors extrapolated from the sample blocks corresponding to the designed random sampling strategies.

The relative error decreases with an increasing number of sampling blocks when the samples are selected across the entire study area. The relative error when selecting 500 blocks is 4.40%. Based on the MODIS-derived proportion of deforestation, the estimation precision improves dramatically if more than 0.1% of the blocks within the regions are sampled. Because the study period is approximately one year, the proportion of deforestation of each block might not be large, so the small areas of deforestation detected with the TM imagery most likely cannot be monitored using MODIS imagery; this changes the variance of the selected samples and results in larger errors when using a larger deforestation proportion for the sample blocks. The random sampling results indicate that the deforestation can be determined with high precision if a suitable MODIS-derived deforestation proportion is used to assist in the sampling and extrapolation from the TM data.

#### 4.2.2. Error Analysis of Stratified Sampling

The relative errors of the extrapolation results from two sample allocation methods, proportionally distributed allocation and Neyman optimal allocation, compared to the TM-derived deforestation map are given in Table 2.

The extrapolation results of the stratified sampling show that the precision of the Neyman optimal allocation method is higher than that of the proportional allocation method. The precisions of the two methods are similar when large numbers of samples, such as 350 and 500 blocks, are used. Furthermore, the precisions of the two stratified sampling methods changed slightly when the number of samples increased from 350 to 500, which indicates that approximately 350 samples are sufficient to obtain a reliable estimate with stratified sampling in this study area.

#### 4.2.3. Error Analysis of Systematic Sampling Estimation

To compare random sampling and stratified sampling with systematic sampling using the same number of samples, we adopted three systematic sampling intervals in which the sample blocks are located at the intersections of the 1.0°, 0.5°, and 0.42° lines of latitude and longitude. These intervals correspond to 83, 355, and 507 sample blocks, respectively. The relative errors are shown in Table 3. The precision with the 0.5° sampling interval is the highest of the three systematic sampling designs. However, because fewer samples are located in strata with a high deforestation probability (>5%), the precision decreases when using 507 samples. These results indicate that both the sample density and the sample locations influence the precision of the deforestation extrapolation.

#### 4.2.4. Comparison of Sampling Strategies

The estimation errors shown in Tables 1, 2, and 3 indicate that stratified sampling with approximately 350 sample blocks, especially for the Neyman optimal allocation method, provides the highest precision of estimates of the total deforestation area in the study area. Random sampling requires 500 samples to achieve the same level of precision. Furthermore, the estimation precision is not stable with a change in the sample density and with few sample blocks. Systematic sampling can obtain more reliable deforestation results than random sampling, which also depends on the sample locations. Sampling blocks with forest cover can provide higher levels of precision for the entire study area. Based on this analysis, stratified sampling is recommended as the best method to combine information from both the low and high spatial resolutions because the low resolution signal allowed for the efficient targeting of deforestation hotspots.

### 4.3. Comparison of Extrapolation Methods

To obtain the spatial distribution of the deforestation sites, a linear regression model between the MODIS-derived and TM-derived deforestation (6) was constructed using the selected sample blocks. The model was then applied to the MODIS-derived deforestation map to obtain the corresponding deforestation area in each block. The deforestation results were evaluated with the TM-derived results, and the relative errors are compared with the results of the simple extrapolation in Table 4.

The relative errors shown in Table 4 lead to several conclusions. First, the regularity of the precision extrapolated from (6) is similar to that obtained with (5). The two extrapolation methods both provide the highest precision with the stratified Neyman optimal allocation sampling method and the lowest precision with random sampling. Second, the precision extrapolated from the regression model is higher than that from (5). The regression model (6) fully utilizes the deforestation data of nonsampled blocks detected from the MODIS dataset, but they are not considered in (5). While only the total deforestation area is retrieved by the simple extrapolation method, the regression extrapolation method can provide the detailed deforestation proportion in each block with higher precision. However, the results estimated from the regression model depend on accurately detected deforestation in both the MODIS and the TM datasets.

## 5. Conclusions

Using the PRODES deforestation data as a reference, deforestation areas are detected from nearly synchronous TM imagery and MODIS datasets. Several sampling designs employing TM-derived and MODIS-derived deforestation were compared to estimate the deforestation across the study area from 2005 to 2006. In general, the sampling approaches merit consideration as timely and cost-effective components for monitoring deforestation over large areas. The complete coverage TM-derived deforestation provides a unique opportunity to assess different sampling designs because it allows comparisons that are based on wall-to-wall estimators and are not estimated from single samples. A stratified sampling method that included strata construction and sample allocation provided more precise estimates than both simple random sampling and systematic sampling. Moreover, regressions between the MODIS-derived and TM-derived deforestation results provide precise estimates of both the total deforestation area and the deforestation distribution in each block.

## Figures and Tables

**Table 1:** Deforestation error *ε* (%) estimated based on random sampling.

Sampling blocks	Random sampling region			
	Study area	>0.1%∗	>0.5%∗	>1%∗
100	29.43	7.28	9.35	31.17
350	13.52	4.76	24.12	29.33
500	4.40	8.13	26.84	34.10

*The proportion means the MODIS-derived deforestation proportion in each block.

**Table 2:** Deforestation error *ε* (%) estimated from two methods of stratified sampling.

Sampling blocks	Proportional allocation	Neyman optimal allocation
100	14.30	8.34
350	4.14	3.74
500	4.04	3.62

**Table 3:** Deforestation error *ε* (%) estimated from systematic sampling.

Strata	One degree interval		0.5 degree interval		0.42 degree interval	
	Blocks in strata (%)^#^	Relative error (%)	Blocks in strata (%)^#^	Relative error (%)	Blocks in strata (%)^#^	Relative error (%)
0-1%	68 (82)	15.02	284 (80)	5.56	405 (80)	7.21
1%–5%	10 (12)		55 (15.5)		86 (17)	
5%–8%	2 (2.4)		10 (2.8)		12 (2.3)	
>8%	3 (3.6)		6 (1.7)		4 (0.7)	
Total	**83 (100)**		**355 (100)**		**507 (100)**	

^#^The number in the parentheses is the proportion of the sample blocks in each stratum.

**Table 4:** Comparison of the relative error *ε* (%) extrapolated from two methods.

Sampling blocks^#^	Random sampling		Stratified proportional allocation		Stratified Neyman optimal allocation		Systematic sampling	
	E1∗	E2∗	E1	E2	E1	E2	E1	E2
100 (83)	29.43	16.51	14.30	9.32	8.34	4.15	15.02	12.14
350 (355)	13.52	12.72	4.14	6.12	3.74	3.36	5.56	6.06
500 (507)	4.40	3.63	4.04	5.16	3.62	2.82	7.21	10.12

^#^The number in the parentheses is the number of sampling blocks used for the systematic sampling.

∗E1 means the simple extrapolation from (5), and E2 is the regression extrapolation from (6) based on the relationship between the MODIS-derived and TM-derived deforestation.
